# [Retracted] Interference with Tim-3 protein expression attenuates the invasion of clear cell renal cell carcinoma and aggravates anoikis

**DOI:** 10.3892/mmr.2025.13766

**Published:** 2025-12-01

**Authors:** Muming Yu, Bin Lu, Yancun Liu, Ying Me, Lijun Wang, Hui Li

Mol Med Rep 15: 1103–1108, 2017; DOI: 10.3892/mmr.2017.6136

Following the publication of the above paper, it was drawn to the Editor's attention by a concerned reader that certain of the cell morphological data shown in Fig. 3 on p. 1106 and the flow cytometric data shown in Fig. 4 on p. 1107 were strikingly similar to data appearing in different form in other articles written by different authors at different research institutes that had already been accepted for publication elsewhere prior to the submission of this paper to *Molecular Medicine Reports*.

In view of the fact that the abovementioned data had already apparently been published previously, the Editor of *Molecular Medicine Reports* has decided that this paper should be retracted from the Journal. The authors were asked for an explanation to account for these concerns, but the Editorial Office did not receive a reply. The Editor apologizes to the readership for any inconvenience caused.

